# Inertial Sensor-Based Smoother for Gait Analysis

**DOI:** 10.3390/s141224338

**Published:** 2014-12-17

**Authors:** Young Soo Suh

**Affiliations:** Department of Electrical Engineering, University of Ulsan, Mugeo, Namgu, Ulsan 680-749, Korea; E-Mail: yssuh@ulsan.ac.kr; Tel.: +82-52-259-2196

**Keywords:** gait analysis, smoother, inertial sensors, inertial navigation algorithm

## Abstract

An off-line smoother algorithm is proposed to estimate foot motion using an inertial sensor unit (three-axis gyroscopes and accelerometers) attached to a shoe. The smoother gives more accurate foot motion estimation than filter-based algorithms by using all of the sensor data instead of using the current sensor data. The algorithm consists of two parts. In the first part, a Kalman filter is used to obtain initial foot motion estimation. In the second part, the error in the initial estimation is compensated using a smoother, where the problem is formulated in the quadratic optimization problem. An efficient solution of the quadratic optimization problem is given using the sparse structure. Through experiments, it is shown that the proposed algorithm can estimate foot motion more accurately than a filter-based algorithm with reasonable computation time. In particular, there is significant improvement in the foot motion estimation when the foot is moving off the floor: the *z*-axis position error squared sum (total time: 3.47 s) when the foot is in the air is 0.0807 m^2^ (Kalman filter) and 0.0020 m^2^ (the proposed smoother).

## 1. Introduction

Gait analysis is the study of human walking [1–3]. It is helpful in the medical diagnosis and treatment of those diseases that affect the ability to walk.

There are many devices that can be used to perform gait analysis [4]. Mechanical goniometers are used to measure hip, knee and ankle angles. Force plates on the floor or on a shoe (such as the wearable force plate from Tec Gihan Co., Kyoto, Japan) are used to measure the vertical force beneath the foot [5]. Optical motion trackers (such as Vicon) are used to measure the movement of a leg and a foot. These sensor systems can be used independently or together to give necessary gait parameters, such as stride length, walking speed and ground reaction forces.

Probably, an optical motion tracker is the most popular gait analysis tool [6], since it provides a very accurate description of foot and leg motion. The disadvantages of an optical motion tracker are its high cost and limited walking range. You can only increased walking range by using additional cameras, which can be quite expensive for a long walking range.

An alternative technology for the optical motion tracker is the use of inertial sensors [7,8]. Although the accuracy of inertial sensor-based estimation is generally worse than that of the optical motion tracker, the inertial sensor approach does not have a walking range limitation, and the cost is generally cheaper. If inertial sensors (gyroscopes and accelerometers) are attached to a leg, the angles of leg joints can be estimated (see [9–11] and commercial products from INSENCO and Xsens). These angle-based systems are extended to estimate whole human body motion [12–14]. Theoretically, the position of a leg also can be estimated using the inertial navigation algorithm [15]. However, the position estimation error quickly diverges when low cost inertial sensors are used. If inertial sensors are attached to a foot, the position (in addition to angle estimation) can be estimated using an inertial navigation algorithm. Unlike in the leg case, we know that the foot touches the ground almost periodically during walking. When the foot touches the ground, the velocity of the foot becomes temporarily zero, and during this zero velocity interval, the velocity errors of the inertial navigation algorithm can be reset. This zero velocity updating significantly reduces the position error [16].

Estimation of foot movement by attaching inertial sensors to the foot has been extensively studied in the context of a pedestrian navigation system [17,18]. In the filter-based foot motion estimation, the position error increases as soon as the foot is off the ground, since there are no measurements when the foot is in the air. The position error decreases once the foot touches the ground from the zero velocity updating. Thus, in the filter-based foot motion estimation, the motion estimation is not accurate when the foot is moving, but the final position (after the zero velocity updating) becomes accurate. In the pedestrian navigation system, the final position of the foot (that is, the final position of a person) is the main concern and, thus, the filter-based estimation can be used.

On the other hand, foot motion trajectory is the main concern in gait analysis. The filter estimated position can be improved using the smoother. The filtering algorithm uses the measurement data up to the current time to compute the current estimation. On the other hand, the smoother uses the whole measurement data (including the future data) to compute the current estimation. In [19], a smoother algorithm is proposed based on the forward-backward filter, where a smoothing algorithm is applied for each walking step. The position after the zero velocity updating is used as the initial value of a backward filter. The smoothed result for each step is combined rather heuristically to produce total walking estimation.

In this paper, the smoothing problem is formulated as a global optimization problem, where the whole walking motion is estimated in a single optimization problem. The smoother algorithm is formulated as a quadratic optimization problem (motivated by [20]), where its problem size depends on the total walking time. Since the problem size becomes very large even for a few minutes of walking data, the efficient solution is given utilizing the sparse structure of the problem.

The remainder of the paper is organized as follows. In Section 2, a basic material is introduced defining the coordinate systems and the main variables. In Section 3, a Kalman filter to estimate the foot motion is given. In Section 4, a smoother algorithm to estimate the foot motion is formulated in the quadratic optimization problem. In Section 5, a solution to the quadratic optimization problem is given using the sparse structure of the problem. In Section 6, experiment results are given to verify the proposed algorithm. Concluding remarks are given in Section 7.

## 2. Problem Formulation

**Figure 1.**
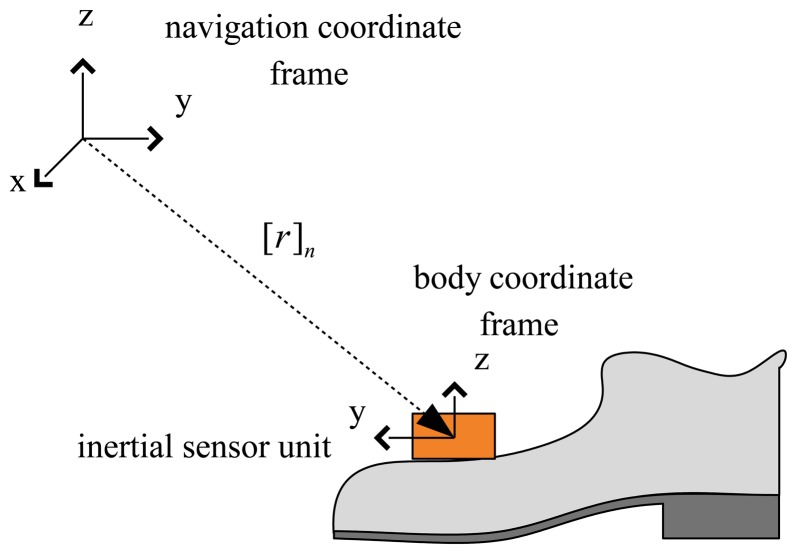
Overview of the foot inertial sensor unit.

Two-coordinate frames (the body coordinate frame and the navigation coordinate frame) are defined as in Figure 1. The three axes of the body coordinate frame coincide with the three axes of the inertial sensor unit. The *z*-axis of the navigation coordinate frame coincides with the local gravitational direction. The choice of the *x*-axis of the navigation coordinate frame can be chosen arbitrarily, since the walking direction is not important in the gait analysis. The notation [*p*]*_n_* ([*p*]*b*) is used to denote that a vector *p* is represented in the navigation (body) coordinate frame.

The position of the foot is defined by [*r*]*_n_* ∈ *R*^3^, which is the origin of the body coordinate frame expressed in the navigation coordinate frame. Similarly, the velocity of the foot is denoted by [*v*]*_n_* ∈ *R*^3^. The attitude of the foot is represented using a quaternion *q* ∈ *R*^4^, which represents the rotation relationship between the navigation coordinate frame and the body coordinate frame. The directional cosine matrix corresponding to quaternion *q* is denoted by *C*(*q*) ∈ *SO*(3).

The basic dynamic equations [21,22] of *q*, *r* and *v* are given by:

(1) $$\begin{array}{l} \dot{q}=\frac{1}{2}\Omega \left(\omega \right)q\\ \dot{v}=C^{\prime }\left(q\right)a_{b}\\ \text{ṙ}=v \end{array}$$

where *ω* = [*ω_x_ ω_y_ ω_z_* ]′ is the angular velocity of the body coordinate frame with respect to the navigation coordinate frame. The symbol Ω(*ω*) is defined by:

$$\Omega \left(\omega \right)≜\left[\begin{array}{cccc} 0 & -\omega_{x} & -\omega_{y} & -\omega_{z}\\ \omega_{x} & 0 & \omega_{z} & -\omega_{y}\\ \omega_{y} & -\omega_{z} & 0 & \omega_{x}\\ \omega_{z} & \omega_{y} & -\omega_{x} & 0 \end{array}\right].$$

The external acceleration (acceleration related to the movement, excluding the gravitational acceleration) expressed in the body coordinate frame is denoted by *a_b_* ∈ *R*^3^.

The goal of this paper is to estimate *q*, *r* and *v*. The estimated values are denoted by *q̂*, *r̂* and *v̂*, respectively The inertial navigation algorithm [19,23] is used to estimate *q*, *r* and *v*, where the equations in Equation (1) are integrated to compute *q̂*, *r̂* and *v̂*. Accelerometer output *y_a_* ∈ *R*^3^ and gyroscope output *y_g_* ∈ *R*^3^ are related to *ω* and *a_b_* as follows:

(2) $$\begin{array}{l} y_{a}=C\left(q\right)\tilde{g}+a_{b}+b_{a}+n_{a}\\ y_{g}=\omega +b_{g}+n_{g} \end{array}$$

where *n_a_* ∈ *R*^3^ and *n_g_* ∈ *R*^3^ are sensor noises. The vector *g̃* is the local gravitational acceleration, where *g̃* = [0 0 9.8] is used. The sensor biases are denoted by *b_a_* ∈ *R*^3^ and *b_g_* ∈ *R*^3^. It is assumed that the sensor biases are constant and unknown.

Let *T* be the sampling period of the sensor output. The sampled outputs of *y_a_* and *y_g_* are denoted by *y_a,k_* = *y_a_*(*kT*) and *y_g,k_* = *y_g_*(*kT*). The same notation is used for *q_k_*, *r_k_* and *v_k_*. Let *N* be the final discrete time index of the sensor output, where the starting index is one. The discrete sensor noise *n_a,k_* and *n_g,k_* are assumed to be zero mean white Gaussian noises, whose covariances are given by:

(3) $$\begin{array}{cc} R_{a}=E\left\{n_{a,k}n_{a,k}^{\prime }\right\}\in R^{3\times 3}, & R_{g}=E\left\{n_{g,k}n_{g,k}^{\prime }\right\}\in R^{3\times 3} \end{array}.$$

Due to high sensor noise level of low cost inertial sensors and unknown biases, an inertial navigation algorithm without external reference measurement can estimate foot motion only for a few seconds. To overcome this problem, a zero velocity updating method is used in an inertial sensor-based foot motion estimation. During walking, the foot touches the ground almost periodically. When the foot is on the ground, we know that the velocity of the foot is zero. Thus, whenever the foot touches the ground, the velocity error in the inertial navigation algorithm can be reset, which significantly reduces foot motion estimation errors. To use this zero velocity updating, zero velocity intervals (that is, when the foot is on the ground) must be detected. Many zero velocity detection algorithms have been proposed [16,24].

In this paper, we use a simple zero velocity detection algorithm. Let *Z_m_* be a set of all discrete time indices belonging to the zero velocity intervals. The discrete time *k* belongs to *Z_m_* if the following conditions are satisfied:

(4) $$\begin{array}{ll} \left\|y_{g,i}\right\|\leq B_{g}, & k-\frac{N_{g}}{2}\leq i\leq k+\frac{N_{g}}{2}\\ \left\|y_{a,i}-y_{a_{i-1}}\right\|\leq B_{a}, & k-\frac{N_{a}}{2}\leq i\leq k+\frac{N_{a}}{2} \end{array}$$

where *N_g_* and *N_a_* are even number integers.

## 3. Kalman Filter

In this section, *q*, *r*, *v*, *b_g_* and *b_a_* are estimated using a Kalman filter. The estimated values are denoted by *q̂_KF_*, *r̂_KF_*, *v̂_KF_*, *b̂_g,KF_* and *b̂_a,KF_*. As in [19,23], *q_k_* ∼ *b_a,k_* are not directly estimated in the Kalman filter. They are first estimated from the integration equation of (1), and then, the errors in the integrated values are estimated in the Kalman filter. These estimated values will be used in Section 4 to derive a smoothed estimation.

### 3.1. Initialization

Since only the relative position is important in the gait analysis, the initial position is assumed to be zero: that is, *r̂_kf_*_,1_ = 0_3×1_, where “1” denotes the initial discrete time index. Assuming that a person is not moving at the initial time, the initial attitude is computed using the triad algorithm [25,26]. The following two vector relations are used in the triad algorithm:

(5) $$\begin{array}{c} {\left[y_{a}\right]}_{b}=C\left({\hat{q}}_{KF,1}\right)\;\left[\begin{array}{c} 0\\ 0\\ 1 \end{array}\right]\\ \left[\begin{array}{c} 1\\ 0\\ 0 \end{array}\right]=C\left({\hat{q}}_{KF,1}\right)\;\left[\begin{array}{c} 1\\ 0\\ 0 \end{array}\right]. \end{array}$$

The pitch and roll angles are determined from the first vector relation, and the yaw angle is determined from the second vector relation. Since the heading of walking is not important in the gait analysis, the yaw angle can be chosen arbitrarily.

The initial velocity is chosen to be zero: that is, *v̂_KF_*_,1_ = 0_3×1_. If there is no particular information on *b_g_* and *b_a_*, the initial estimates are chosen to be zero (*b̂*_*g,KF*,1_ = *b̂*_*a,KF*,1_ = 0_3×1_).

### 3.2. Integration of Estimated Values

Suppose we have computed *q̂_KF,k_*, *r̂_KF,k_*, ⋯ , *b̂_a, KF,k_* at the discrete time *k.* The estimated values at the discrete time *k* + 1 are firstly computed using Equation (1), where *ω* and *a_b_* are replaced by *y_g_* − *b̂_g_* and *y_a_* − *C*(*q̂*)*g̃* − *b̂_a_*:

(6) $$\begin{array}{l} \dot{\hat{q}}=\frac{1}{2}\Omega \left(y_{a}-{\hat{b}}_{a}\right)\hat{q}=\frac{1}{2}\Omega \left(\omega +n_{g}-{\hat{b}}_{g}\right)\hat{q}\\ \dot{\hat{v}}=C{\left(\hat{q}\right)}^{\prime }\left(y_{a}-{\hat{b}}_{a}\right)-\tilde{g}=C{\left(\hat{q}\right)}^{\prime }\left(a_{b}+n_{a}+b_{a}-{\hat{b}}_{a}\right)+\left(C{\left(\hat{q}\right)}^{\prime }C\left(\hat{q}\right)-I\right)\tilde{g}\\ \dot{\hat{r}}=\hat{v}. \end{array}$$

For later use, we define a function *f_k_*, which represents a numerical integration of Equation (6) from *kT* to *(k* + 1)*T*:

(7) $$\left[\begin{array}{c} {\hat{q}}_{KF,k+1}\\ {\hat{r}}_{KF,k+1}\\ {\hat{v}}_{KF,k+1} \end{array}\right]=f_{k}\;\left(\left[\begin{array}{c} {\hat{q}}_{KF,k}\\ {\hat{r}}_{KF,k}\\ {\hat{v}}_{KF,k} \end{array}\right],\left[\begin{array}{c} n_{g,k}\\ n_{a,k} \end{array}\right],\left[\begin{array}{c} b_{g}-{\hat{b}}_{g,k}\\ b_{a}-{\hat{b}}_{a,k} \end{array}\right]\right).$$

We assume that the variables not used as arguments of *f_k_* are embedded in *f_k_*: for example, the *ω*, *C*(*q*) and *g̃* terms are assumed to be embedded in the function *f_k_*.

### 3.3. Kalman Filter

Due to noise terms *(n_g,k_* and *n_a,k_*) and bias estimation error (*b_g_* − *b̂_g,k_* and *b_a_* − *b̂_a,k_*), there are errors in the numerically-integrated values of Equation (7). These errors are estimated using a Kalman filter. Let *X_KF,k_* be defined by:

(8) $$X_{KF,k}=\left[\begin{array}{c} {\bar{q}}_{KF,k}\\ {\bar{r}}_{KF,k}\\ {\bar{v}}_{KF,k}\\ {\bar{b}}_{g,KF,k}\\ {\bar{b}}_{a,KF,k} \end{array}\right]=\left[\begin{array}{c} \left[\begin{array}{cc} 0_{3\times 1} & I_{3} \end{array}\right]\left({\hat{q}}_{KF,k}^{*}⊗q_{k}\right)\\ r_{k}-{\hat{r}}_{KF,k}\\ v_{k}-{\hat{v}}_{KF,k}\\ b_{g}-{\hat{b}}_{g,KF,k}\\ b_{a}-{\hat{b}}_{a,KF,k} \end{array}\right]\in \left[\begin{array}{c} R^{3}\\ R^{3}\\ R^{3}\\ R^{3}\\ R^{3} \end{array}\right]$$

where ⊗ is the quaternion multiplication and q* denotes the quaternion conjugate of a quaternion *q* [21]. The elements of *X_KF,k_* represent the estimation errors in *q̂_KF,k_* ∼ *b̂_a,KF,k_*. In defining *X_KF,k_*, it is assumed that the quaternion errors in *q̂_KF,k_* are small. Thus, (*q̂_KF,k_*)* ⊗ *q_k_* is approximated by:

$$\left({\hat{q}}_{KF,k}\right)*⊗q_{k}\approx \left[\begin{array}{c} 1\\ {\bar{q}}_{KF,k} \end{array}\right]\in \left[\begin{array}{c} R\\ R^{3} \end{array}\right].$$

With this assumption, the error in *q̂_KF,k_* ∈ *R*^4^ is represented by three-dimensional vector *q̄_KF,k_* instead of a four-dimensional vector. See [27] for the details about the multiplicative error notation of a quaternion. The initial estimate is set to *X̂_KF_*_,1_ = 0_15×1_, and the initial error covariance *P*_1_ is given by:

(9) $$\begin{array}{c} P_{1}=E\left\{\left(X_{KF,1}-{\hat{X}}_{KF,1}\right){\left(X_{KF,1}-{\hat{X}}_{KF,1}\right)}^{\prime }\right\}\\ =\left[\begin{array}{ccccc} P_{q,\text{init}} & 0_{3\times 3} & 0_{3\times 3} & 0_{3\times 3} & 0_{3\times 3}\\ 0_{3\times 3} & P_{r,\text{init}} & 0_{3\times 3} & 0_{3\times 3} & 0_{3\times 3}\\ 0_{3\times 3} & 0_{3\times 3} & P_{v,\text{init}} & 0_{3\times 3} & 0_{3\times 3}\\ 0_{3\times 3} & 0_{3\times 3} & 0_{3\times 3} & P_{b_{g,\text{init}}} & 0_{3\times 3}\\ 0_{3\times 3} & 0_{3\times 3} & 0_{3\times 3} & 0_{3\times 3} & P_{b_{a,\text{init}}} \end{array}\right] \end{array}.$$

The initial attitude error covariance *P_q,init_* is given by the algorithm in [25], where *R_a_* and 10^−^^11^*I*_3_ are used as measurement noise covariances of the first and second equations in Equation (5). The small covariance value for the second equation is to make *P_q,init_* nonsingular, since $P_{q,\text{init}}^{-1}$

is used in the smoothing algorithm. The remaining covariances *P_r,init_* ∼ *P_ba,init_* can be considered as design parameters, whose values are given in Table 1.

The dynamic equation of *X_KF,k_* is given by:

(10) $$X_{KF,k+1}=[\begin{array}{cc} \Phi_{k+1,k} & \Psi_{k+1,k}\\ 0_{9\times 6} & I_{6} \end{array}]X_{KF,k}+\left[\frac{w_{k}}{0_{6\times 1}}\right]$$

where:

(11) $$e^{A_{k}T}=\left[\begin{array}{cc} \Phi_{k+1,k} & \Psi_{k+1,k}\\ 0_{9\times 6} & I_{6} \end{array}\right]\in [\begin{array}{cc} R^{9\times 9} & R^{9\times 6}\\ R^{6\times 9} & R^{6\times 6} \end{array}]$$

$$A_{k}=\left[\begin{array}{cc} \begin{array}{ccc} -\left[y_{a,k}\times \right] & 0_{3\times 3} & 0_{3\times 3}\\ 0_{3\times 3} & 0_{3\times 3} & I_{3}\\ -2C{\left({\hat{q}}_{KF,k}\right)}^{\prime }\left[y_{a,k}\times \right] & 0_{3\times 3} & 0_{3\times 3} \end{array} & \begin{array}{cc} -\frac{1}{2}I_{3} & 0_{3\times 3}\\ 0_{3\times 3} & 0_{3\times 3}\\ 0_{3\times 3} & -C{\left({\hat{q}}_{KF,k}\right)}^{\prime } \end{array}\\ \begin{array}{ccc} \;0_{6\times 3} & \;0_{6\times 3} & 0_{6\times 3} \end{array} & \begin{array}{cc} 0_{6\times 3} & 0_{6\times 3} \end{array} \end{array}\right]=\left[\begin{array}{cc} A_{k,1} & A_{k,2}\\ 0_{6\times 9} & 0_{6\times 6} \end{array}\right].$$

For a vector *p* = [*p*_1_ *p*_2_ *p*_3_]′ ∈ *R*^3^, [*p*×] is defined by:

$$\left[p\times \right]=\left[\begin{array}{ccc} 0 & -p_{3} & p_{2}\\ p_{3} & 0 & -p_{1}\\ -p_{2} & p_{1} & 0 \end{array}\right].$$

Note that *A_k_* is a lower triangular matrix, and thus, *e^AkT^* is also a lower triangular matrix having the structure in Equation (11) [28].

The process noise *w_k_* is the zero mean white Gaussian noise with:

$$Q_{k}=E\left\{w_{k}w_{k}^{\prime }\right\}=\int_{0}^{T}\exp\left(A_{k,1}r\right)\;\left[\begin{array}{ccc} \frac{1}{4}R_{g} & 0_{3\times 3} & 0_{3\times 3}\\ 0_{3\times 3} & 10^{-6}I_{3} & 0_{3\times 3}\\ 0_{3\times 3} & 0_{3\times 3} & R_{a} \end{array}\right]\exp\left(A_{k,1}^{\prime }r\right)dr$$

where the 10^−^^6^ value in *Q_k_* is added to make *Q_k_* nonsingular, since $Q_{k}^{-1}$ is used in the smoother. Derivation of *A_k_* and *Q_k_* can be obtained by slightly modifying the results in [19,23]. The computational load of *e^AkT^* can be reduced by using the following approximation:

(12) $$e^{A_{k}T}\approx I_{15}+A_{k}T+\frac{1}{2!}A_{k}^{2}T^{2}.$$

#### Remark 1

*How the estimation error in k-th discrete time evolves after the integration Equation* (7) *is represented in Equation* (10). *For example*, (10) *states that the error in r̂_KF,k_*_+1_ *comes from two sources. One is from the estimation error in k-th time (before the integration), and the other is from the process of numerical integration. The latter is not from the numerical integration error (it is ignored), but from the noise terms and bias estimation errors*.

In an inertial sensor-only foot motion estimation, there is no external measurement. The fact that the velocity is zero during the zero velocity intervals is used as a fictitious measurement.

During the zero velocity interval, the following equation can be used as a measurement equation:

(13) $$0_{3\times 1}-{\hat{v}}_{KF,k}=\left[\begin{array}{ccccc} 0_{3\times 3} & 0_{3\times 3} & I_{3} & 0_{3\times 3} & 0_{3\times 3} \end{array}\right]\;X_{KF,k}+n_{r,k},k\in Z_{m}$$

where *n_v,k_* is a fictitious measurement noise representing the confidence of zero velocity interval detection in Equation (4). The *z*-axis value of *r̂_KF,k_* is almost the same when the foot is on the ground, assuming that a person is walking on a flat floor. Thus, the following measurement equation can also be used during the zero velocity interval.

(14) $$0-\left[\begin{array}{ccc} 0 & 0 & 1 \end{array}\right]\;{\hat{r}}_{KF,k}=\left[\begin{array}{ccccc} 0_{1\times 3} & \begin{array}{ccc} 0 & 0 & 1 \end{array} & 0_{1\times 3} & 0_{1\times 3} & 0_{1\times 3} \end{array}\right]\;X_{KF,k}+n_{r,k},k\in Z_{m}$$

where *n_r,k_* is a fictitious measurement noise.

We assume that *n_v,k_* and *n_r,k_* are zero mean white Gaussian noises, where covariances are given by:

(15) $$\begin{array}{cc} R_{v}=\text{E}\left\{n_{v,k}n_{v,k}^{\prime }\right\}\in R^{3\times 3}, & R_{r}=\text{E}\left\{n_{r,k}n_{r,k}^{\prime }\right\}\in R. \end{array}$$

A standard discrete time Kalman filter is applied to Equations (10), (13) and (14). If *k* ∈ *Z_m_*, we can compute *X̂_KF,k_* from the Kalman filter, and these values are used to update *q̂_KF,k_* ∼ *b∈_a,KF,k_*.

The Kalman filter algorithm of position estimation (*r̂_KF,k_*) is summarized in the following for *k*_1_ ≤ *k* ≤ *k*_2_ assuming that *k*_1_ ∈ *Z_m_*, *k*_2_ ∈ *Z_m_* and *k* ∉ *Z_m_* for *k*_1_ < *k* < *k*_2_. The algorithm for other values *(q̂_KF,k_*, *v̂_KF,k_, b̂_g,KF,k_* and *b̂_a,KF,k_*) are similar.

- discrete time *k* = *k*_1_ ∈ *Z_m_*
  – *r̂_KF,k_* is given from the previous step
  – *r̄̂_KF,k_* = 0
- discrete time *k*_1_ < *k* < *k*_2_
  – compute *r̂_KF,k_* using Equation (7)
  – *r̄̂_KF,k_*_−__1_ = 0 from Kalman filter time update Equation (10) with *r̄̂_KF,k_*_−__1_ = 0
- discrete time *k* = *k*_2_ ∈ *Z_m_*
  – compute *r̂_KF,k_* using Equation (7)
  – compute *r̄̂_KF,k_* from the Kalman filter measurement update Equations (13) and (14)
  – *r̂_KF,k_* ⟸ *r̂_KF,k_* + *r̄̂_KF,k_*
  – *r̄̂_KF,k_*_−__1_ = 0

The result in this section is not a new result, and similar results can be found in [19].

## 4. Smoothing Algorithm

In this section, the Kalman filter-estimated values (*q̂_KF,k_* ∼ *b̂_a,KF,k_*) in Section 3 are improved using the smoother, where the errors in *q̂_KF,k_*, *r̂_KF,k_*, *v̂_KF,k_, b̂_g,KF_* and *b̂_a,KF_* are compensated for. To do that, the estimation error is defined as follows.

(16) $$\begin{array}{l} {\bar{q}}_{SM,k}=\left[\begin{array}{cc} 0_{3\times 1} & I_{3} \end{array}\right]{\hat{q}}_{KF,k}^{*}⊗q_{k}\\ {\bar{r}}_{SM,k}=r_{k}-{\hat{r}}_{KF,k}\\ {\bar{v}}_{SM,k}=v_{k}-{\hat{v}}_{KF,k}\\ {\bar{b}}_{g,SM}=b_{g}-{\hat{b}}_{g,KF}\\ {\bar{b}}_{a,SM}=b_{a}-{\hat{b}}_{a,KF} \end{array}$$

where *b̂_g,KF_* and *b̂_a,KF_* are *b̂_g,KF,N_* and *b̂_a,KF,N_* (the final values) in the Kalman filter, which can be considered as the most accurate estimation of *b_g_* and *b_a_*. Since *b_g_* and *b_a_* are constant, *b̄_g,SM_* and *b̄_a,SM_* are not time dependent, while *q̄_SM,k_*, *r̄_SM,k_* and *v̄_SM,k_* are time dependent. We note that the estimation errors in Equation (8) represent the errors before the measurement update, while the errors in Equation (16) represent the errors after the measurement update.

Let *X_SM,k_* be defined by:

(17) $$X_{SM,k}=\left[\begin{array}{c} {\bar{q}}_{SM,k}\\ {\bar{r}}_{SM,k}\\ {\bar{v}}_{SM,k} \end{array}\right].$$

Equations for time-dependent variables (*q̄_SM,k_*, *r̄_SM,k_* and *v̄_SM,k_*) are given by:

(18) $$\zeta_{k}+X_{SM,k+1}=\Phi_{k+1,k}X_{SM,k}+\psi_{k+1,k}\left[\begin{array}{c} {\bar{b}}_{g,SM}\\ {\bar{b}}_{a,SM} \end{array}\right]+w_{k}$$

where Φ and Ψ are defined in Equation (11) and:

(19) $$\zeta_{k}=\left[\begin{array}{c} {\tilde{q}}_{SM,k}\\ {\hat{r}}_{KF,k+1}-f_{2,k}\\ {\hat{v}}_{KF,k+1}-f_{3,k} \end{array}\right],$$

(20) $$\begin{array}{c} \left[\begin{array}{c} f_{k,1}\\ f_{k,2}\\ f_{k,3} \end{array}\right]=f_{k}\;\left(\left[\begin{array}{c} {\hat{q}}_{KF,k}\\ {\hat{r}}_{KF,k}\\ {\hat{v}}_{KF,k} \end{array}\right],\left[\begin{array}{c} n_{g,k}\\ n_{a,k} \end{array}\right],\left[\begin{array}{c} {\bar{b}}_{g,SM}\\ {\bar{b}}_{a,SM} \end{array}\right]\right),\\ {\tilde{q}}_{SM,k}=\left[\begin{array}{cc} 0_{3\times 1} & I_{3} \end{array}\right]\;f_{1,k}^{*}⊗{\hat{q}}_{KF,k+1}. \end{array}$$

Derivation of Equation (18) is now explained. From Equations (16), (19) and (20), we have:

(21) $$\zeta_{k}+X_{SM,k+1}=\left[\begin{array}{c} \left[\begin{array}{cc} 0_{3\times 3} & I_{3} \end{array}\right]\;f_{1,k}^{*}⊗q_{k+1}\\ r_{k+1}-f_{2,k}\\ v_{k+1}-f_{3,k} \end{array}\right]\in R^{9}.$$

The second and the third elements of Equation (21) are straightforward. The first element of Equation (21) is derived from the following:

(22) $$\begin{array}{ll} q_{k+1} & ={\hat{q}}_{KF,k+1}⊗\;\left[\begin{array}{c} 1\\ {\bar{q}}_{SM,k+1} \end{array}\right]\\ & =f_{1,k}⊗f_{1,k}^{*}⊗{\hat{q}}_{KF,k+1}⊗\;\left[\begin{array}{c} 1\\ {\bar{q}}_{SM,k+1} \end{array}\right]\\ & \approx f_{1,k}⊗\;\left[\begin{array}{c} 1\\ {\tilde{q}}_{SM,k}+{\bar{q}}_{SM,k+1} \end{array}\right] \end{array}$$

where the following approximation is used:

(23) $$f_{1,k}^{*}⊗{\hat{q}}_{KF,k+1}\approx \left[\begin{array}{c} 1\\ {\tilde{q}}_{SM,k+1} \end{array}\right],$$

(24) $$\left[\begin{array}{c} 1\\ {\tilde{q}}_{SM,k} \end{array}\right]\;⊗\;\left[\begin{array}{c} 1\\ {\bar{q}}_{SM,k+1} \end{array}\right]\approx \left[\begin{array}{c} 1\\ {\tilde{q}}_{SM,k}+{\bar{q}}_{SM,k+1} \end{array}\right].$$

From Equation (21), *ξ_k_* + *X_SM,k_*_+1_ denotes the estimation errors of *f_k_* in Equation (20). Thus, Equation (18) represents how the estimation error evolves after the integration Equation (20). Thus, Equation (18) is essentially the same as Equation (10). In the exact same methods in the case of Equation (10), Equation (21) can be obtained by slightly modifying the results in [19,23].

The smoothing problem is formulated as a quadratic optimization problem using the method in [20]. Let an optimization variable *X̃* be defined by:

(25) $$\tilde{X}=\left[\frac{\begin{array}{c} X_{SM,1}\\ X_{SM,2}\\ \vdots \\ X_{SM,N} \end{array}}{\begin{array}{c} {\bar{b}}_{g,SM}\\ {\bar{b}}_{a,SM} \end{array}}\right]\in \left[\frac{R^{9\times N}}{R^{6}}\right].$$

The smoothing problem can be formulated as the following quadratic optimization problem:

(26) $$\underset{\tilde{X}}{\min}J\left(\tilde{X}\right)$$

where:

(27) $$\begin{array}{c} J\left(\tilde{X}\right)=\frac{1}{2}{\text{∑}}_{k=1}^{N-1}w_{k}^{\prime }Q_{k}^{-1}w_{k}+\frac{1}{2}{\text{∑}}_{k\in Z_{m}}{\left(z_{k}-{\tilde{H}}_{k}X_{SM,k}\right)}^{\prime }{\tilde{R}}^{-1}\left(z_{k}-{\tilde{H}}_{k}X_{SM,k}\right)\\ +\frac{1}{2}{\left(X_{SM,1}-X_{\text{init}}\right)}^{\prime }P_{X_{\text{init}}}^{-1}\left(X_{SM,1}-X_{\text{init}}\right)\\ +\frac{1}{2}{\left({\hat{b}}_{g,SM}+{\bar{b}}_{g,SM}-b_{g,\text{init}}\right)}^{\prime }P_{b_{g,\text{init}}}^{-1}\left({\hat{b}}_{g,SM}+{\bar{b}}_{g,SM}-b_{g,\text{init}}\right)\\ +\frac{1}{2}{\left({\hat{b}}_{a,SM}+{\bar{b}}_{a,SM}-b_{a,\text{init}}\right)}^{\prime }P_{b_{a,\text{init}}}^{-1}\left({\hat{b}}_{a,SM}+{\bar{b}}_{a,SM}-b_{a,\text{init}}\right) \end{array}$$

$$X_{\text{init}}=\left[\begin{array}{c} {\hat{q}}_{KF,1}\\ 0_{3\times 3}\\ 0_{3\times 1} \end{array}\right],P_{X_{\text{init}}}=\left[\begin{array}{ccc} P_{q,\text{init}} & 0_{3\times 3} & 0_{3\times 3}\\ 0_{3\times 3} & P_{r,\text{init}} & 0_{3\times 3}\\ 0_{3\times 3} & 0_{3\times 3} & P_{v,\text{init}} \end{array}\right]$$

$$z_{k}=\left[\begin{array}{c} 0_{3\times 1}-{\hat{v}}_{KF,k}\\ 0-\left[\begin{array}{ccc} 0 & 0 & 1 \end{array}\right]{\hat{r}}_{KF,k} \end{array}\right],{\tilde{H}}_{k}=\left[\begin{array}{ccc} 0_{3\times 3} & 0_{3\times 3} & I_{3}\\ 0_{1\times 3} & \begin{array}{ccc} 0 & 0 & 1 \end{array} & 0_{1\times 3} \end{array}\right],\tilde{R}=\left[\begin{array}{cc} R_{v} & 0\\ 0 & R_{r} \end{array}\right].$$

Initial information on *b_g_* and *b_a_* are reflected in *b_g,init_* ∈ *R*^3^ and *b_a,init_* ∈ *R*^3^. For example, *b_g,init_* can be computed if a sensor unit is put on the ground without moving for 1∼2 min. If there is no information on *b_g_* and *b_a_*, we set *b_g,init_* and *b_a,init_* to zero.

Inserting Equation (18) into Equation (27), we have:

(28) $$\begin{array}{c} J\left(\tilde{X}\right)=\frac{1}{2}{{\text{∑}}_{k=1}^{N-1}\left(\zeta_{k}+X_{SM,k+1}-\Phi_{k+1,k}X_{SM,k}-\Psi_{k+1,k}\left[\begin{array}{c} {\bar{b}}_{g,SM}\\ {\bar{b}}_{a,SM} \end{array}\right]\right)}^{\prime }Q^{-1}\\ \left(\zeta_{k}+X_{SM,k+1}-\Phi_{k+1,k}X_{SM,k}-\Psi_{k+1,k}\left[\begin{array}{c} {\bar{b}}_{g,SM}\\ {\bar{b}}_{a,SM} \end{array}\right]\right)\\ +\frac{1}{2}{\text{∑}}_{k\in Z_{m}}{\left(z_{k}-{\tilde{H}}_{k}X_{SM,k}\right)}^{\prime }R^{-1}\left(z_{k}-{\tilde{H}}_{k}X_{SM,k}\right)+\frac{1}{2}{\left(X_{SM,1}-X_{\text{init}}\right)}^{\prime }P_{X_{\text{init}}}^{-1}\left(X_{SM,1}-X_{\text{init}}\right)\\ +\frac{1}{2}{\left({\hat{b}}_{g,SM}+{\bar{b}}_{g,SM}-b_{g,\text{init}}\right)}^{\prime }P_{b_{g,\text{init}}}^{-1}\left({\hat{b}}_{g,SM}+{\bar{b}}_{g,SM}-b_{g,\text{init}}\right)\\ +\frac{1}{2}{\left({\hat{b}}_{a,SM}+{\bar{b}}_{a,SM}-b_{a,\text{init}}\right)}^{\prime }P_{b_{a,\text{init}}}^{-1}\left({\hat{b}}_{a,SM}+{\bar{b}}_{a,SM}-b_{a,\text{init}}\right). \end{array}$$

It is not difficult to see that Equation (28) is a quadratic function of *X̃* ∈ *R*^9×^*^N^*^+6^:

(29) $$J\left(\tilde{X}\right)=\frac{1}{2}\tilde{X}\prime M_{1}\tilde{X}+M_{2}^{\prime }\tilde{X}+M_{3}$$

where *M*_1_, *M*_2_ and M_3_ can be computed from Equation (28). *M*_1_ and *M*_2_ are given in the Appendix (*M*_3_ is omitted, since it is irrelevant in the optimization). The minimizing solution of Equation (29) can be computed by solving the following linear equation:

(30) $$M_{1}{\tilde{X}}^{*}+M_{2}=0$$

where X̃* is the minimizing solution.

Once the minimizing solution X̃* is computed, the smoother estimated values are computed using Equation (16). For example, the position estimate is computed as follows:

$${\hat{r}}_{SM,k}={\hat{r}}_{KF,k}+X_{SM,k}^{*}$$

where *r̂_SM,k_* is the smoother estimate of *r_k_* and $X_{SM,k}^{*}$ is the 9(*k* − 1) + 1 ∼ 9*k* rows of X̃*.

The result in this section is a new result. The main contribution of this section is that the foot motion Kalman filter error is compensated for using the quadratic optimization problem.

## 5. Smoothing Optimization Solution

Although the smoothing problem boils down to a simple linear Equation (30)*M*_1_ ∈ *R*^(9*N*+6)×(9*N*+6)^ becomes very large when *N* is large. Suppose we solve Equation (30) using the Cholesky decomposition. Let *L* be the Cholesky triangle (lower triangular matrix) satisfying:

(31) $$M_{1}=LL\prime .$$

Once *L* is computed, X̃* can be computed by the following equation:

(32) $$\begin{array}{l} L\tilde{Y}=-M_{2}\\ L\prime {\tilde{X}}^{*}=\tilde{Y}. \end{array}$$

The computational load of Cholesky decomposition Equation (31) is (9*N* + 6)^3^/6 flops, and the load for each linear equation in Equation (32) is (9*N* + 6)^2^/6 flops [29]. Suppose *T* = 0.01 and *N* = 12,000 (2-min walk data), then the computational load of the Cholesky decomposition alone is approximately 2 × 10^14^ flops, which is practically difficult to compute (due to the very long computation time and large memory requirement).

Fortunately, the matrix *M*_1_ has a sparse structure, and the computational load can be reduced using the sparse structure.

We partition *M*_1_ and *M*_2_ as follows:

(33) $$M_{1}=\left[\frac{M_{11}}{M_{12}^{\prime }}|\frac{M_{12}}{M_{13}}\right]\in \left[\frac{R^{9N\times 9N}}{R^{6\times 9N}}|\frac{R^{9N\times 6}}{R^{6\times 6}}\right],M_{2}=\left[\frac{M_{21}}{M_{22}}\right]\in \left[\frac{R^{9N\times 1}}{R^{6\times 1}}\right].$$

We also partition *X̃* in Equation (29) accordingly:

(34) $$\tilde{X}=\left[\frac{{\tilde{X}}_{1}}{{\tilde{X}}_{2}}\right]\in \left[\frac{R^{9N\times 1}}{R^{6\times 1}}\right]$$

From the definition of *M*_11_ in the Appendix, we can see that *M*_11_ is a banded matrix [29] with the bandwidth *p* = 17:

(35) $$M_{11}=\left[\begin{array}{ccccccc} {}* & * & & & & & \\ {}* & * & * & & & & \\ & * & * & * & & & \\ & & \ddots & \ddots & \ddots & & \\ & & & * & * & * & \\ & & & & * & * & *\\ & & & & & * & * \end{array}\right]$$

where * denotes the arbitrary 9 × 9 block matrix. All unspecified entries are zero 9 × 9 block matrices.

Equation (30) can be rewritten using the partitions in Equations (33) and (34):

(36) $$\left[\frac{M_{11}}{M_{12}^{\prime }}|\frac{M_{12}}{M_{13}}\right]\;\left[\frac{{\tilde{X}}_{1}}{{\tilde{X}}_{2}}\right]=-\left[\frac{M_{21}}{M_{22}}\right]$$

Equation (36) can be transformed into the following equation using the Schur complement [30]:

(37) $$\begin{array}{cc} \left(M_{13}-M_{12}^{\prime }M_{11}^{-1}M_{12}\right)\;{\tilde{X}}_{2} & =M_{22}-M_{12}^{\prime }M_{11}^{-1}M_{21}\\ M_{11}{\tilde{X}}_{1} & =M_{21}-M_{12}{\tilde{X}}_{2}. \end{array}$$

The Equation (37) can be solved in the following sequence [30]. First, find the Cholesky decomposition of banded matrix *M*_11_:

(38) $$M_{11}=L_{11}L_{11}^{\prime }$$

where L_11_ ∈ *R*^9^*^N×^*^9^*^N^* is a lower triangular matrix. Find *U*_1_ ∈ *R*^9^*^N^*^×1^ and *U*_2_ ∈ *R*^9^*^N^*^×1^ from the following linear equation:

(39) $$\begin{array}{l} L_{11}U_{1}=M_{12}\\ L_{11}U_{2}=M_{21}. \end{array}$$

Inserting Equation (39) into Equation (37), we have:

(40) $$\left(M_{13}-U_{1}^{\prime }U_{1}\right){\tilde{X}}_{2}=M_{22}-U_{1}^{\prime }U_{2}.$$

After computing *X̃*_2_ in Equation (40), we compute *X̃*_1_ from the following equations:

(41) $$\begin{array}{l} L_{11}U_{3}=M_{21}-M_{12}{\tilde{X}}_{2}\\ L_{11}^{\prime }{\tilde{X}}_{1}=U_{3}. \end{array}$$

When solving Equations (38)–(41), the algorithms for banded matrices in [29] can be used for efficient computation and memory usage. The computational load of the Cholesky decomposition of the banded matrix Equation (38) is (9*N*(*p*^2^ + 3*p*))/2 flops (*p* = 17 is the bandwidth of *M*_11_, and 9*N* ≫ *p* is assumed) and 9*N* square root computation. The computational load of each equation in Equations (39) and (41) is 9*N*(*p* + 1) flops. Equation (40) is a small dimensional problem (*X̃*_2_ ∈ *R*^6^).

Compared with the standard method, the solution using the banded structure of *M*_11_ requires much smaller computation and memory. For example, the Cholesky decomposition requires approximately 3.6 × 10^7^ flops and 9*N* square root computation in the case of *N* = 12, 000. Compared with 2 × 10^14^ flops in the standard computation case, its computational requirement is significantly small.

The main contribution of this section is to derive an efficient solution to the quadratic optimization problem and to investigate the quantitative computational requirement.

## 6. Experiment

**Figure 2.**
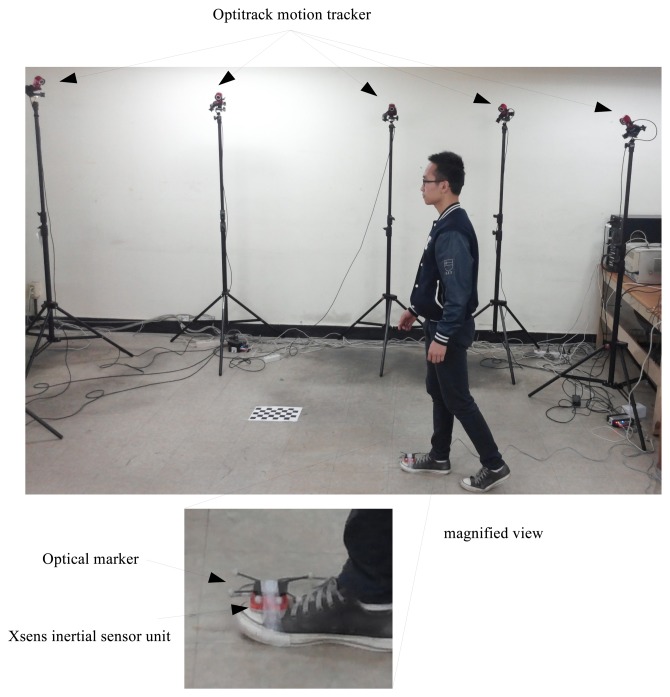
Experimental setup for the first experiment.

Two experiments are performed to verify the proposed algorithm. In the experiments, an inertial sensor unit (Xsens MTi sensor unit) is attached on the foot, as shown in Figure 2. The parameters used in both experiments are given in Table 1.

In the first experiment, a person walked three steps, while the movement of the foot is tracked using an optical motion tracking system (Optitrack six Flex 13 camera system). The experimental setup is given in Figure 2. Six optical markers are used in the optical tracking system, and the center position of six markers is used as the true foot position. Since the coordinate systems of the optical tracker and the inertial sensor unit are different, the optical tracker output is rotated and translated.

**Figure 3.**
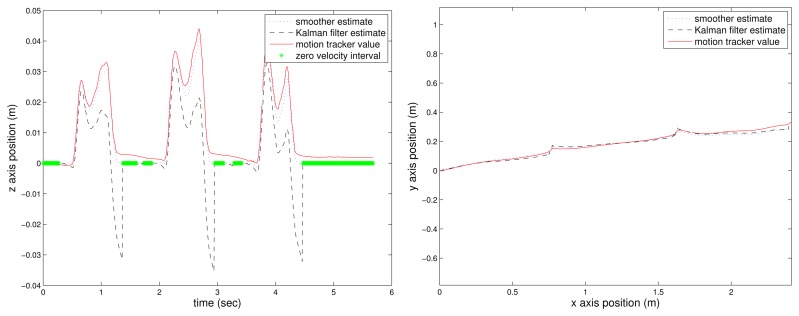
Estimated position with the flat floor assumption: *z*-axis position **(Left)** and *xy*-axis position **(Right).**

The estimated position (Kalman filter and the proposed smoother) with the flat floor assumption (see Equation (14)) is given in Figure 3: the left figure is the *z*-axis movement, and the right figure is the *xy*-axis position. In the left figure, the zero velocity intervals are represented by the “*” symbol.

Assuming that the motion tracker value is the true position value, we can see that the Kalman filter gives a good final position estimation (around 5 s). However, the position estimated during walking is not accurate; for example, see the values between 2 and 3 s. Thus, the Kalman filter result may be good enough for the pedestrian navigation system, where the final position is important. However, the Kalman result is not good enough for gait analysis, where it is important to know how the foot has moved in addition to the final position. The final estimated position of the smoother is almost similar to that of the Kalman filter. However, we can see that there is significant improvement in the middle foot position estimation.

The *z*-axis estimation error is compared quantitatively in Table 2. The *z*-axis estimation error *e_z,k_* is defined by:

$$e_{z,k}=\left[\begin{array}{ccc} 0 & 0 & 1 \end{array}\right]\;\left(r_{k}-{\hat{r}}_{k}\right)$$

where *r̂_k_* is either *r̂_k_* = *r̂_KF,k_* or *r̂_k_* = *r̂_SM,k_*.

The *z*-axis estimation error squared sum is given in Table 2. The estimation error of both algorithms during the zero velocity interval (*k* ∈ *Z_m_*) are almost the same, since the estimated values becomes zero with the flat floor assumption. The estimation error when the foot is in the air are different; the proposed smoother gives significantly smaller estimation error: 0.0020 in the case of the proposed smoother and 0.0807 in the case of the Kalman filter. Furthermore, the maximum z-axis position errors for the Kalman filter and the proposed smoother are 4 cm and 0.5 cm, respectively. This improved estimation is due to the fact the smoother uses all of the available information.

**Figure 4.**
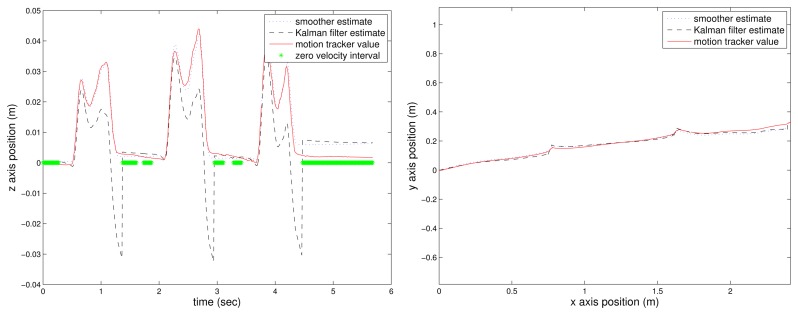
Estimated position without the flat floor assumption: *z*-axis position **(Left)** and *xy*-axis position **(Right).**

The position estimation by the Kalman filter and the proposed smoother without the flat floor assumption are given in Figure 4. Without the flat floor assumption, we can see that the *z*-axis error increases since the *z*-axis position error is not corrected using Equation (14).

Due to the viewpoint constraint of the optical tracker, the walking distance is rather limited in the first experiment. In the second experiment, a person walked 50 m along a straight line on the flat floor. In this experiment, there is no ground truth value, except the *z*-axis value, which should be almost constant during the zero velocity intervals, since a person walked on a flat floor.

**Figure 5.**
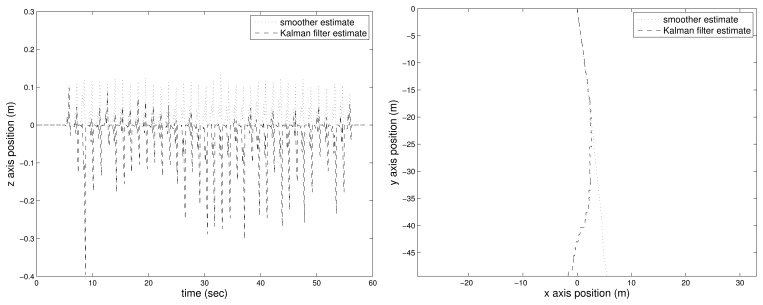
Estimated position (50-m walk): z-axis position **(Left)** and *xy*-axis position **(Right).**

The Kalman filter estimated and smoother estimated positions are given in Figure 5, both with the flat floor assumption. In the Kalman filter result, the estimated *xy* trajectory is slightly curved. The smoother estimated *xy* trajectory is a little bit more straight. The true trajectory should be almost a straight line, since the person walked along a straight line.

Similar to the first experiment, there is no significant improvement in the smoother position estimation for the position where the foot is on the floor (that is, after the zero velocity updating). The smoother, however, gives more accurate foot motion estimation while the foot is moving in the air, which is important in the gait analysis. This can be seen in the *z*-axis graphs: the Kalman filter-estimated *z*-axis trajectory is not accurate, since the *z*-axis value cannot be negative (which means the foot is beneath the floor).

The 50-m walk is repeated by four subjects: five times for each person, thus a total of 20 experiments. The total walk length estimation is given in Table 3. The average estimated walk length is 48.99 m with the standard deviation being 0.26 m. From the small standard deviation, the estimation result does not seem to depend on the subjects.

We note that the *xy*-axis position error increases as the walk length increases. This is unavoidable, since the motion is estimated using the inertial navigation algorithm without any external reference. The *z*-axis position error increment is limited with the flat floor assumption.

The computation time of the proposed smoother was 8.7 s (MATLAB running on Intel Core i5-2310 CPU Windows PC, 2.9 GHz), where the walk time is 58.92 s (*N* = 5892).

## 7. Conclusions

In this paper, a smoother algorithm is proposed for gait analysis to obtain more accurate foot motion estimation than the filter-based estimation. In the smoother algorithm, the motion estimation problem is formulated as a quadratic optimization problem, and an efficient computational algorithm is given.

In the first experiment, we have compared the results of the Kalman filter and the proposed smoother. The Kalman filter-based estimation is quite accurate after the zero velocity update. However, foot motion estimation is not accurate when the foot is in the air. We have shown that the motion estimation is significantly improved for the foot motion in the air if the smoother is used: the *z*-axis position error squared sum when the foot is in the air is 0.0807 (Kalman filter) and 0.0020 (the proposed smoother). Thus, the proposed smoother is a useful algorithm for gait analysis, where the foot motion estimation is important.

In the second experiment, we investigated a long walk foot motion estimation (50 m). There is not much difference in the final position estimation of the Kalman filter and the proposed smoother. The 50-m walk was tested using four subjects, and we found that the estimation does not depend on the subject. This is not surprising, since there are no person-dependent parameters in the algorithm.

Since the proposed smoother algorithm is more complicated than the Kalman filter, the computational time is slow. In the second experiment, the computation time of the proposed smoother is about 8.7 s to process about one minute of walk data (the sampling period is 0.01 s), while it took 0.8 s for the Kalman filter. Thus, the estimation performance is improved with the additional computation time.

## Figures and Tables

**Table 1.** Parameters of the proposed smoother and Kalman filter.

**Parameter**	**Symbol**	**Values**	**Related Equation**
sampling period	T	0.01	
zero velocity detection parameters	*B_g_*, *N_g_*, *B_a_*, *N_a_*	0.2, 10, 0.4, 10	(4)
sensor noise error covariance	*R_g_, R_a_*	0.001*I*_3_, 0.01*I*_3_	
measurement noise covariance	*R_v_*, *R_r_*	0.0001*I*_3_,0.0001	(15)
filter and smoother parameter	*P_r,init_*, *P_v,init_*	0.0001*I*_3_, 0.0001*I*_3_	(9), (27)
filter and smoother parameter	*P_bg,init_*, *P_ba,init_*	0.000001*I*_3_, 0.000001*I*_3_	(9), (27)

**Table 2.** Estimated *z* position error squared sum with the flat floor assumption.

**Algorithms**	**$E_{1}={\text{∑}}_{k\in Z_{m}}e_{z,k}^{2}$**	**$E_{2}={\text{∑}}_{k\notin Z_{m}}e_{z,k}^{2}$**	***E*_1_ + *E*_2_**
	(error squared sum when the foot is on the ground)	(error squared sum when the foot is in the air)	
Kalman filter	0.0008	0.0807	0.0815
Proposed smoother	0.0009	0.0020	0.0029

**Table 3.** Four persons' 50-m walk experiment results.

**Subject Number**	**Final *xy* Position Estimated Value (m)**	**Average (m)**
1	48.97, 48.97, 49.16, 49.06, 49.02	49.04
2	49.36, 49.29, 49.21, 49.37, 49.14	49.27
3	48.52, 48.98, 48.46, 48.69, 48.45	48.62
4	48.98, 49.00, 48.92, 49.08, 49.06	49.01

## A. Appendix

In this Appendix, *M*_11_, *M*_12_, *M*_13_, *M*_21_, *M*_22_ (partition of M_1_ and *M*_2_ in Equation (33)) are defined. We use the notation *M*_11_[*i*, *j*], which represents a *R*^9×9^ matrix extracting 9(*i* − 1) + 1 ∼ 9*i* rows and 9(*j* − 1) + 1 ∼ 9*j* columns of *M*_11_. Similarly, we use *M*_12_[*i*] ∈ *R*^9×6^ and *M*_21_[*i*] ∈ *R*^9×1^ notations.

- *M*_11_
  $$\begin{array}{c} M_{11}\left[k,k\right]=\{\begin{array}{ll} P_{X_{\text{init}}}^{-1}+\Phi_{21}^{\prime }Q_{k}^{-1}\Phi_{21} & k=1\\ \Phi_{k+1,k}^{\prime }Q_{k}^{-1}\Phi_{k+1,k}+Q_{k}^{-1}, & 2\leq k\leq N-1\\ Q_{k}^{-1}, & k=N. \end{array}\\ M_{11}\left[k,k\right]=M_{11}\left[k,k\right]+{\tilde{H}}_{k}^{\prime }{\tilde{R}}^{-1}{\tilde{H}}_{k},k\in Z_{m}\\ \begin{array}{cc} M_{11}\left[k,k+1\right]=-\Phi_{k+1,k}^{\prime }Q_{k}^{-1}, & 1\leq k\leq N-1 \end{array}\\ \begin{array}{cc} M_{11}\left[k+1,k\right]=-Q_{k}^{-1},\Phi_{k+1,k}, & 1\leq k\leq N-1 \end{array} \end{array}$$
- *M*_12_
  $$M_{12}\left[k\right]=\{\begin{array}{ll} \Phi_{k+1,k}^{\prime }Q_{k}^{-1}\Psi_{k+1,k}, & k=1\\ \Phi_{k+1,k}^{\prime }Q_{k}^{-1}\Psi_{k+1,k}-\Psi_{k,k-1}, & 2\leq k\leq N-1\\ -\Psi_{k,k-1}, & k=N. \end{array}$$
- *M*_13_
  $$M_{13}=\left(\overset{N-1}{\underset{k=1}{\text{∑}}}\Psi_{k+1,k}^{\prime }Q_{k}^{-1}\Psi_{k+1,k}\right)+\left[\begin{array}{cc} P_{b_{g,\text{init}}}^{-1} & 0_{3\times 3}\\ 0_{3\times 3} & P_{b_{a,\text{init}}}^{-1} \end{array}\right]$$
- *M*_21_
  $$M_{21}\left[k\right]=\{\begin{array}{ll} -\zeta_{k}^{\prime }Q_{k}^{-1}\Phi_{k+1,k}, & k=1\\ -\zeta_{k}^{\prime }Q_{k}^{-1}\Phi_{k+1,k}+\zeta_{k-1}^{\prime }Q_{k}^{-1}, & 2\leq k\leq N-1 \end{array}$$
- *M*_22_
  $$M_{22}=\left(\overset{N-1}{\underset{k=1}{\text{∑}}}-\zeta_{k}^{\prime }\Psi_{k+1,k}\right)+\left[\begin{array}{cc} {\left({\hat{b}}_{g,SM}-b_{g,\text{init}}\right)}^{\prime }P_{b_{g,\text{init}}}^{-1} & 0\\ 0 & {\left({\hat{b}}_{a,SM}-b_{a,\text{init}}\right)}^{\prime }P_{b_{a,\text{init}}}^{-1} \end{array}\right]$$
